# Image-Based Wheat Fungi Diseases Identification by Deep Learning

**DOI:** 10.3390/plants10081500

**Published:** 2021-07-21

**Authors:** Mikhail A. Genaev, Ekaterina S. Skolotneva, Elena I. Gultyaeva, Elena A. Orlova, Nina P. Bechtold, Dmitry A. Afonnikov

**Affiliations:** 1 Institute of Cytology and Genetics, Siberian Branch of the Russian Academy of Sciences, 630090 Novosibirsk, Russia; mag@bionet.nsc.ru (M.A.G.); skolotnevaES@bionet.nsc.ru (E.S.S.); 2 Faculty of Natural Sciences, Novosibirsk State University, 630090 Novosibirsk, Russia; 3 Kurchatov Genomics Center, Institute of Cytology and Genetics, Siberian Branch of the Russian Academy of Sciences, 630090 Novosibirsk, Russia; 4 All Russian Institute of Plant Protection, Pushkin, 196608 St. Petersburg, Russia; eigultyaeva@gmail.com; 5 Siberian Research Institute of Plant Production and Breeding, Branch of the Institute of Cytology and Genetics, Siberian Branch of Russian Academy of Sciences, 630501 Krasnoobsk, Russia; orlova.lena10@yandex.ru (E.A.O.); Telichkinanina@mail.ru (N.P.B.)

**Keywords:** wheat, leaf rust, powdery mildew, septoria, stem rust, yellow rust, image recognition, deep learning, convolutional neural network, phenotyping

## Abstract

Diseases of cereals caused by pathogenic fungi can significantly reduce crop yields. Many cultures are exposed to them. The disease is difficult to control on a large scale; thus, one of the relevant approaches is the crop field monitoring, which helps to identify the disease at an early stage and take measures to prevent its spread. One of the effective control methods is disease identification based on the analysis of digital images, with the possibility of obtaining them in field conditions, using mobile devices. In this work, we propose a method for the recognition of five fungal diseases of wheat shoots (leaf rust, stem rust, yellow rust, powdery mildew, and septoria), both separately and in case of multiple diseases, with the possibility of identifying the stage of plant development. A set of 2414 images of wheat fungi diseases (WFD2020) was generated, for which expert labeling was performed by the type of disease. More than 80% of the images in the dataset correspond to single disease labels (including seedlings), more than 12% are represented by healthy plants, and 6% of the images labeled are represented by multiple diseases. In the process of creating this set, a method was applied to reduce the degeneracy of the training data based on the image hashing algorithm. The disease-recognition algorithm is based on the convolutional neural network with the EfficientNet architecture. The best accuracy (0.942) was shown by a network with a training strategy based on augmentation and transfer of image styles. The recognition method was implemented as a bot on the Telegram platform, which allows users to assess plants by lesions in the field conditions.

## 1. Introduction

**Figure 1:**
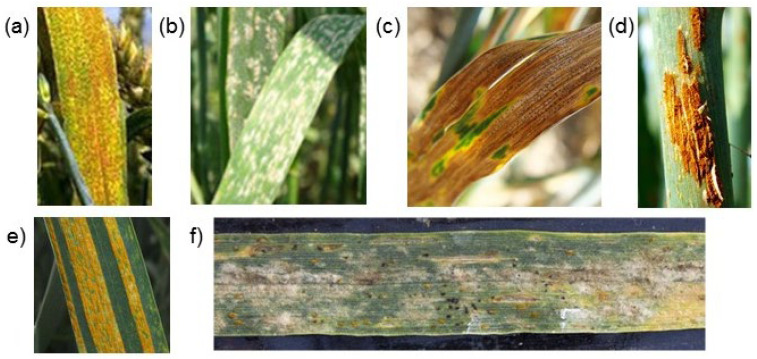
Examples of digital images showing manifestations of wheat plant disease: (**a**) leaf rust, (**b**) powdery mildew, (**c**) septoria, (**d**) stem rust, (**e**) yellow rust, and (**f**) multiple diseases.

Wheat is one of the world’s main crops and food sources for human consumption [1]. Wheat accounts for the largest planting area, and the food security of the population of most countries of the world depends on its yield. One of the main factors affecting wheat yield is fungi diseases: rust, septoria of leaves and ears, and powdery mildew [2] (Figure 1).

Leaf rust, powdery mildew, and septoria (pathogens *Puccinia triticina* Erikss., *Blumeria graminis* (DC.) Speer, *Zymoseptoria tritici* Rob., and *Parastaganospora nodorum* Berk.) are cosmopolitan pathogens. They are observed in the phytopathogenic complex on grain crops widely. However, the total damage caused by the causative agents of the above-listed diseases does not exceed 20% and is controlled by the timely use of fungicides [3]. The consequences of the epiphytoties of yellow and stem rust, leading to grain shortages of more than 30%, are of serious economic importance. In general, grain yield losses from these diseases, depending on the region and season conditions, can vary from 15 to 30% or more [4,5]. The most effective way to combat these diseases is their prevention and timely implementation of protective actions [4,6,7]. However, such a strategy is impossible without a timely and correct diagnosis of pathogens. In this case, it is important to identify diseases at the stage of seedlings, since at later stages of plant development, resistance to the pathogen is higher [8]. In turn, the effectiveness of such diagnostics largely depends on how accurate, labor-intensive, and resource-intensive they are.

Visual assessment of the diseases has been the main diagnostic method throughout the history of wheat cultivation. It allows to identify plaque, pustules, spots, or necrosis [9,10]. It requires the training of specialists in phytopathology and a lot of routine work, in addition to the observations (keeping a record book, statistical processing of observations, etc.). In recent decades, molecular, spectral methods, and methods based on the analysis of digital images have appeared and have become widespread [11,12]. These approaches differ in the labor intensity, as well as in its cost and accuracy. The methods, using the analysis of digital RGB images, are based on determining changes in the color, texture, and shape of plant organs that arise as a result of changes in their pigment composition under the influence of the vital activity of pathogens [13,14,15,16,17]. The advantages of such methods are the low cost of monitoring equipment (it is sufficient to use a digital camera or a mobile phone) and the high monitoring speed. The disadvantages include low sensitivity (in comparison, for example, with spectral methods; see References [12,18]).

Recently the technologies for plant-disease monitoring based on digital RGB images have received a powerful impulse due to the improvement of machine learning methods based on the use of neural network algorithms. A feature of deep learning neural networks in comparison with other methods is the multilayer architecture of neurons, in which the next layer uses the output of the previous layer as input data to derive ideas regarding the analyzed objects [19,20]. For example, for such an important task as image labeling, some of the most successful methods are convolutional neural networks (CNNs), for which several architecture options are used. Among the first types of CNN architecture were AlexNet [21] and VGG [22]. Further development of these approaches made it possible to improve the convergence of the proposed algorithms (ResNet network) [23], reduce the number of parameters due to deep convolution (MobileNet network) [24], and improve model training results due to adaptive recalibration of responses across channels (SENet network) [25]. These advances have expanded the applicability of deep learning neural networks. Moreover, they have also demonstrated exceptional success on complex problems of plant phenotyping [19,26,27,28,29,30].

The deep learning neural networks were implemented efficiently in disease detection for various plant species [31,32,33,34,35,36,37]. It was demonstrated that deep learning approaches outperform machine learning algorithms, such as support vector machine, random forest, stochastic gradient descent [38]. The development of the deep learning methods towards better disease recognition in plants included transfer learning approaches [39], implementing networks of various architectures [37,40,41,42,43,44], working with the limited amount of data [45], and Bayesian deep learning [46].

A number of deep learning methods, which have been developed to identify wheat diseases by using digital RGB images, have proved to be effective. In the work by Barbedo [47], CNN of the GoogLeNet architecture was used to detect lesions in the leaf image, and, on this basis, wheat diseases, such as blast, leaf rust, tan spot, and powdery mildew were identified. In the work by Picon et al. [48], a method was developed for identifying four types of wheat diseases, taking into account their stage of development (septoria, tan spot, and two types of rust) based on deep CNNs. Lu et al. [49] have developed an automatic system for diagnosing six types of wheat diseases that recognizes the type of disease and localizes the lesions in the image, obtained in the field conditions.

The increase in the use of deep learning architectures provide significant progress in the diagnosis of wheat diseases based on the digital images. However, there are still gaps to be investigated regarding the use of especially new deep learning architectures in wheat leaf fungal disease detection.

First of all, to build a successful algorithm for disease recognition, a set of a large number of labeled images is required [50]. The availability of such data in the public domain is the basis for improving modern methods of image recognition for plant phenotyping [51,52] and identification of pathogens [53].

Second, progress in disease recognition depends largely on the choice of neural network architecture [37]. New and modified deep learning architectures are constantly being introduced to better/transparent plant disease detection [37]. Improved versions of state-of-the-art models tend to provide high accuracy in disease detection [31,54]. Recently, the EfficientNet network architecture was proposed in Reference [55], and it has shown high efficiency in image labeling. It uses a new activation function called Swish instead of the Rectifier Linear Unit (ReLU) activation function implemented in other CNN models. EfficientNet is a family of CNNs of similar architecture (B0 to B7) which differ from each other in the depth of the layers, their width, and the size of the input image, while maintaining the ratios between these sets of parameters. Thus, as the model number grows, the number of calculated parameters does not increase much. On the ImageNet task, the EfficientNet-B7 model with 66 M parameters achieved an accuracy of 84.3% [55]. This architecture was used for the fruit recognition and demonstrated higher performance compared to the ConvNet architecture [29]. Atila et al. demonstrated that EfficientNet architecture outperforms AlexNet, ResNet, VGG16, and Inception V3 networks in the plant-disease recognition task on the PlantVillage dataset [42]. Zhang et al. [41] implemented EfficientNet for greenhouse cucumber-disease recognition. They demonstrated that the EfficientNet-B4 model outperforms AlexNet, VGG16, VGG19, Inception V4, ResNet50, ResNet101, SqueeseNet, and DenseNet networks.

Another difficulty is the simultaneous presence of symptoms caused by different diseases [56]. On the one hand, the problem of multiple classifications is difficult to solve, since it requires a larger volume of images for correct classification, in which various combinations of lesions will be presented in sufficient numbers. On the other hand, the visual manifestations of each of the diseases can be very similar (see Figure 1), which makes it difficult to make a correct classification.

Finally, one of the important conditions for creating a recognition method is the possibility of using it in field conditions [57]. This increases the efficiency of disease monitoring and, consequently, the chances of successful plant treatment by fungicides. One of the approaches is using mobile devices for this, both for semi-automatic determination of the degree of plant damage [58] and fully automatic analysis, including computer vision methods [59,60] and deep learning networks [48,49,61,62].

Here we propose a method for the recognition of five fungi diseases of wheat shoots (leaf rust, stem rust, yellow rust, powdery mildew, and septoria), both separately and in combination, with the possibility of identifying the stage of plant development. Our paper makes the following contributions:

- The Wheat Fungi Diseases (WFD2020) dataset of 2414 wheat images for which expert labeling was performed by the type of disease. In the process of creating this set, a data-redundancy reduction procedure was applied based on the image hashing algorithm.
- The disease-recognition algorithm based on the use of a network with the EfficientNet-B0 architecture with transfer learning from ImageNet dataset and augmentation, including style transfer.
- The implementation of the recognition method as a chatbot on the Telegram platform, which allows for the assessment of plants’ symptoms in the field conditions.

The structure of our paper is as follows. Section 2.1 provides an overview of our work. The dataset preparation and its description are given below in Section 2.2, Section 2.3, Section 2.4 and Section 3.1, Section 3.2, Section 3.3, respectively. The network architecture and evaluation methods are described in Section 2.5, Section 2.6, Section 2.7; the accuracy estimation results are presented in Section 3.4. A description of the application structure is given in Section 2.8, and a brief description of the functionality is in Section 3.5. We summarize our contribution and compare our results with results obtained by other authors in the Discussion section.

## 2. Materials and Methods

### 2.1. Methods Overview

**Figure 2:**
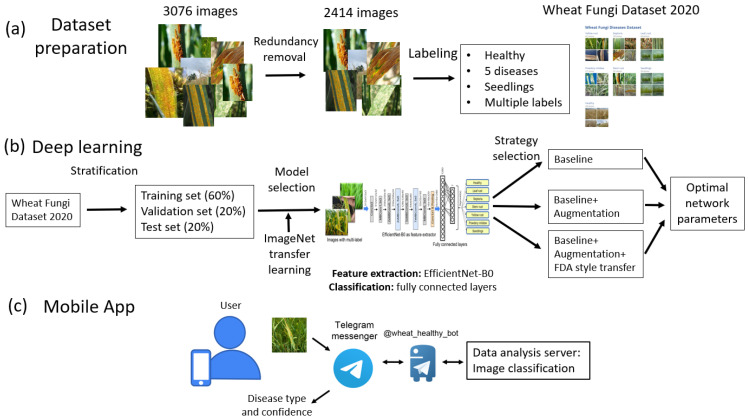
Overview of the approaches developed in this work for wheat fungi diseases recognition: (**a**) image dataset preparation, (**b**) deep learning neural network design and training, and (**c**) mobile application for plant monitoring using Telegram chatbot.

The summary of the methods used in this work is shown in Figure 2.

First, we prepared the Wheat Fungi Diseases image dataset (Figure 2a).

Second, we searched for the optimal parameters of the EfficientNet-B0-based neural network, using the transfer learning technique for initial settings and choosing the best of three training strategies (Figure 2b).

Third, we developed a mobile application for wheat-plant disease detection based on Telegram messenger chatbot technology (Figure 2c).

### 2.2. Image Dataset

The images were obtained from various sources. Some images were taken from the dataset presented at the machine learning challenge “ICLR Workshop Challenge # 1: CGIAR Computer Vision for Crop” on the Zindi.africa platform from 29 January 2020 to 29 March 2020 [63]. Some images were obtained from the plant disease detection platform (PDDP) dataset [61,64]. Some images were taken from the Internet using the Google Images service, and the other part of the dataset was obtained by the authors of the current work by plant imaging in laboratory and field conditions.

### 2.3. Filtration of Images

A preliminary analysis of the original set of images showed that, among them, there were completely duplicated images of the same object with different levels of compression, resolution, or brightness. All of these factors lead to redundancy of information in the training data, which could affect the training process of the classification method and its testing. It was decided to compose a non-redundant set of unique images based on the initial data. To do this, the hashing algorithm aHash was used, implemented in the python ImageHash library [65], the average_hash() method. This algorithm returns a hash value for an image file, a 64-bit integer that does not change when scaling, changing the aspect ratio and slightly changing the contrast and brightness of the image. All initial images were grouped by hash function value. If the group included more than one image, the image from the file with the largest size was selected as its representative. In particular, 612 images were excluded from the original Zindi.africa dataset, which included 1486 images.

A description of the number of image files from various sources is presented in Table 1. It includes data from the Zindi.africa project, the Platform for Plant Disease Detection (PDDP), images obtained through the Google Images service, images obtained by the authors in field and laboratory conditions in 2016–2020 in St. Petersburg, and images obtained by the authors in field and laboratory conditions in 2017–2020 in the Novosibirsk Region.

### 2.4. Labeling of Images

Each image in the WFD2020 dataset was manually annotated. The images were classified into healthy plants (label “healthy”) and by six types of fungal diseases: leaf rust, powdery mildew, septoria, stem rust, and yellow rust. Additionally, the images were classified according to the stage of plant development: whether the plant is a seedling (label “seedling”) or not (absence of such a label). Images could be tagged with several labels at the same time. This occurred when the plant was affected by several diseases or was a seedling.

### 2.5. Neural Network Structure

**Figure 3:**
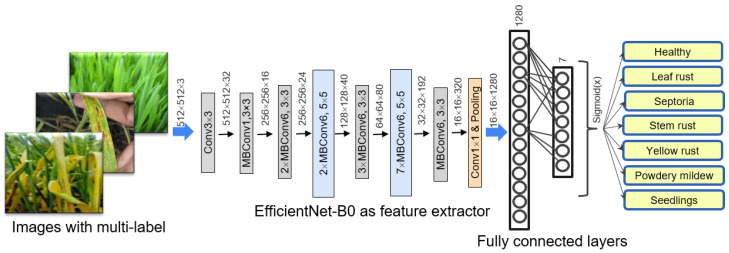
Diagram of the neural network used to predict fungal diseases of wheat.

Here we used the EfficientNet-B0 architecture, which is the simplest and fastest among EfficientNet family [55]. Its structure is shown in Figure 3, and the layers are described in detail in Table 2. It includes seven types of sequential blocks built on the basis of the Conv and MBConv layers [66]. In MBConv, blocks consist of a layer that first expands and then compresses the channels, so direct connections are used between bottlenecks that connect much fewer channels than expansion layers. This architecture has in-depth separable convolutions that reduce calculation compared to traditional layers [24].

The EfficientNet-B0 network structure is implemented by using the PyTorch v1.7.1 [67] and catalyst v20.10.1 [68] frameworks. We used transfer learning technique in our work: the initial values of the network weights dataset were taken from the EfficientNet_PyTorch repository [69]. These weights were obtained by training network on the ImageNet dataset.

In order to implement the classification of images converted by using the EfficientNet-B0 network, two fully connected layers were added to the head of the network: 1280 nodes for the inner layer and 7 nodes for the output layer (according to the number of predicted label types). The Dropout (0.5) regularization was applied to the hidden fully connected layer, which randomly zeroes out half of the layer weights. As a loss function, we used a combination of the activation function Sigmoid(x) and the binary cross entropy BCEWithLogitsLoss(), implemented in the nn module of the PyTorch library. The type of label in the final implementation of the network was determined by the threshold value of the corresponding neuron. If this value was greater than 0.5, then the image was considered to contain the corresponding label. Thus, the image could be marked with several labels at the same time.

### 2.6. Evaluation Scenarios and Performance Assessment

For machine learning, the images were split into 3 subsamples: training 1454 (60%) images, which were used to train the model; validation 480 (20%), images for choosing the best model during training; and test/hold out 480 (20%) images to assess the accuracy of the selected model. To ensure a balanced distribution of multi-label classes in each subsample, the iterative stratification algorithm was used, implemented in the iterative_train_test_split () method of the model_selection python module of the skmultilearn library [70].

For each image, the network predicted 7 numbers, each of which characterized the presence or absence of a particular label. To assess the accuracy of the method on a sample of test images, we compared the predicted set of such numbers and the true set for each image, so if the test sample contained *M* images, we made *7 × M* of such binary comparisons and based on them calculated the true positive (TP) values, true negative values (TN), as well as the total number of positive (1, P) and negative (0, N) values. Based on these values, at the end of each epoch, the accuracy (ACC) value (catalyst.metrics.accuracy.multilabel_accuracy() method) was calculated for images from the validation set according to the formula ACC = (TP + TN)/(P + N). Additionally, we calculated *F1*-score [20] for various labels.

### 2.7. Training Strategies

To optimize the network parameters in the training process using the stochastic gradient descent method, the Adam algorithm was used (implemented in the optim module of the PyTorch library [67]) with the regularization parameter weight_*decay* = 1 × 10^−6^ and the initial learning rate *lr* = 1 × 10^−4^. The learning rate varied according to the CosineAnnealingWarmRestarts heuristic (implemented in the optim.lr_scheduler package of the PyTorch library) with the parameters *T_0* = 10, *T_mult* = 2, *eta_min* = 1 × 10^−6^. The learning was performed by 150 epochs; the batch size was 16. The network was trained on RGB images transformed to 512 × 512 px, while maintaining proportions.

Three training strategies were considered.

(1). Basic strategy EfficientNet-B0_baseline. Training the model by using the above-described methods without any additional modifications to the data and training parameters.

(2). EfficientNet-B0_augm strategy with augmentation. The model was trained by using the above-described methods with the introduction of random noise in the loss of 1%, according to the label-smoothing method [71]. Image augmentation was used by the algorithms implemented in the Albumentations library [72]. Transformations to images from the training sample were applied with a probability of 0.3 and included: rescaling by using the method IAAPerspective (*scale* = (0.02, 0.05)); random rotation ±15 degrees ShiftScaleRotate (*rotate_limit* = (−15.0, 15.0)); vertical and horizontal reflections by using the HorizontalFlip() and VerticalFlip() methods; changing brightness and contrast by using the RandomBrightnessContrast(*brightness_limit* = 0.2, *contrast_limit* = 0.2) method, as well as random masking of square areas by using the Cutout() method [73].

(3) The strategy with augmentation and transfer of image styles EfficientNet-B0_FDA. This strategy used transformations as in the EfficientNet-B0_augm strategy with an additional transformation of the style transfer using the FDA algorithm [74], the FDA() method of the domain_adaptation package of the Albumentations library with the mask parameter *β* = 0.01.

One of the options for using the style transfer method in the learning process is to choose a style from a randomly selected sample image. However, our preliminary analysis has shown that the approach is more effective when several typical styles are first identified for a sample, characteristic images are selected for these styles, and their style is used in the learning process.

To highlight typical styles in the sample, 1000 random images were selected from it. For each image, the Fast Fourier Transform (FFT) method of the fft2() package of the fft numpy library was used. For the resulting transformation, the ifftshift() method was applied, which shifts the low-frequency components of the spectrum, carrying information about the image style, to the center of the spectrum. After that, the elements of the central part of the amplitude component of the spectrum were extracted with a size of 30×30. Thus, for each image, we obtained 900 characteristics, the amplitude components of the low-frequency part of the spectrum. The t-SNE method (t-distributed stochastic neighbor embedding) [75] was applied to this data to isolate two components. After that, the images were clustered in the space of two components by the *k*-means method (*k* = 10). For each cluster, the image closest to the centroid was determined, which represented the corresponding type of image style. Thus, 10 source images were selected to transfer their style to other images in the training process.

We used Grad-CAM (Gradient-weighted Class Activation Map) algorithm [76] from TorchCAM package [77] for the visualization of the network activation map. This technique assigns each neuron a relevance score for the class prediction in the output layer. Grad-CAM backpropagates this information to the last convolutional layer.

A computer with a GPU Nvidia RTX 2080ti was used to train the models. Depending on the type of the applied transformations, the training time for one epoch ranged from 1 to 1.5 min. Based on the results of the training process, the optimal values of the network parameters were determined by the epoch for which the maximum values of the ACC metric were achieved, calculated on the images of the validation sample. We ran our tasks more than 25 times (including preliminary evaluation of models and strategies), which took about 90 h of computer load in total.

### 2.8. Telegram Chatbot

To implement the user interface, we used the chatbot in the Telegram messenger. The chatbot is a virtual Telegram user who can exchange messages with other users, using the Telegram bot Application Program Interface (API). Virtual users access the network and communicate through the same channels available to the users [78,79]. The main task of the bot is an automatic response after the command entered by the user. In our case, the communication is simple. First, the user uploads the plant image taken by smartphone camera via bot interface to the application server. Then the server classifies the user’s image and reports the result to the user via chatbot interface. The chatbot interface was used for several reasons. First, the messenger provides all the major functionalities (image uploading, analysis report) which are accessible via simple API calls. Second, it is much faster to develop and much easier to maintain the chatbot interface in comparison with standalone mobile app. Third, the system works on all mobile and desktop platforms without the need to build system-specific versions.

**Figure 4:**
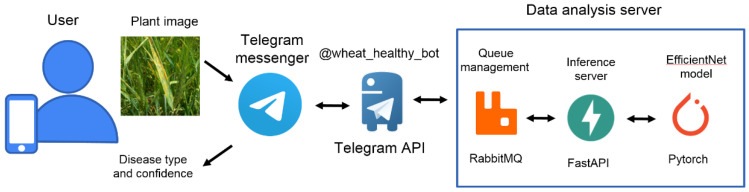
The implementation of the Telegram chatbot for prediction of the wheat fungal diseases. From left to right: user inputs plant image via Telegram messenger; @wheat_healthy_bot provides interface for the data analysis server by using Telegram API; Queue manager controls the Inference server load; Inference server performs wheat image classification using EfficientNet neural network model.

The overview of the chatbot implementation is shown in Figure 4. The user can send the bot single image or an album of images. After receiving the images, @wheat_healthy_bot upload them to the server for analysis. The server part of the system includes three main components. Inference server accepts a request with a user’s image, runs the recognition of the fungal disease by using the EfficientNet model, and sends the results back to the bot. Queue managing system balance the inference server load. The Queue managing system was implemented by using the RabbitMQ [80]; the inference server was implemented by using the FastAPI framework [81]; the EfficientNet model was implemented by using PyTorch [67]. The aiotg library [82] was used for interaction with the Telegram API.

## 3. Results

### 3.1. Wheat Fungi Diseases Dataset

The original image dataset included 3076 images. As a result of image filtering, a non-redundant set of 2414 color images of wheat in jpeg format (Wheat Fungi Diseases dataset, WFD2020) was obtained. These images comprise 78% of the initial dataset. Thus, more than 20% of the redundant images were removed. The total size of the image files was 4.84 GB. The average image resolution was approximately 1500 × 1200 pixels.

**Figure 5:**
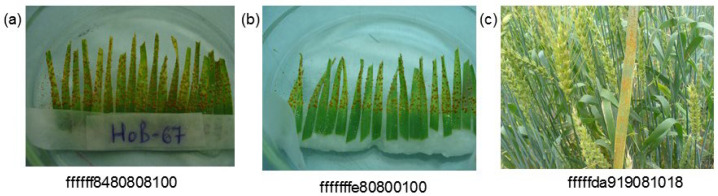
Examples of images of wheat leaves and shoots affected by leaf rust. Below the image, there are the file names (hash function values), which are similar for similar images (**a**,**b**) but differ significantly for different images (**a**,**c**) and (**b**,**c**).

The WFD2020 dataset used for network training is available for public access at the website http://wfd.sysbio.ru/ (accessed on 16 July 2021). All images can be downloaded from the site in the form of a zip archive; expert annotation for all images is available in csv format for annotations for training, validation, and training subsamples that were used in the process of training the models. The images with a label of a certain class can be viewed on the website. The filename for the images matches the value of aHash. Since the hash values for similar images calculated are close in Hamming distance, similar images will be in files with similar names, and to search for similar images, it is sufficient to sort them by the file name. An example of three images of which two are similar, and the third one differs from them, along with the values of the hash functions, is shown in Figure 5.

### 3.2. Results of Labeling a Set of Images

As a result of annotation, the images from the dataset have 3147 labels, resulting in 1.30 labels per image, on average. The number of occurrences of labels of each class in the dataset is presented in Table 3. It could be seen that the label which is most represented in the dataset comprises 20% of the all labels (leaf rust). The label with the smallest fraction (septoria) is about one-quarter of the most frequent label. We may conclude that our dataset is moderately balanced.

Table 4 shows the number of occurrences of various combinations of labels in the WFD2020 dataset. These data demonstrate that more than 80% of images corresponds to single disease labels (including seedlings), more than 12% represented by healthy plants and 6% of the images labeled by multiple diseases.

### 3.3. Clustering Images by Style

**Figure 6:**
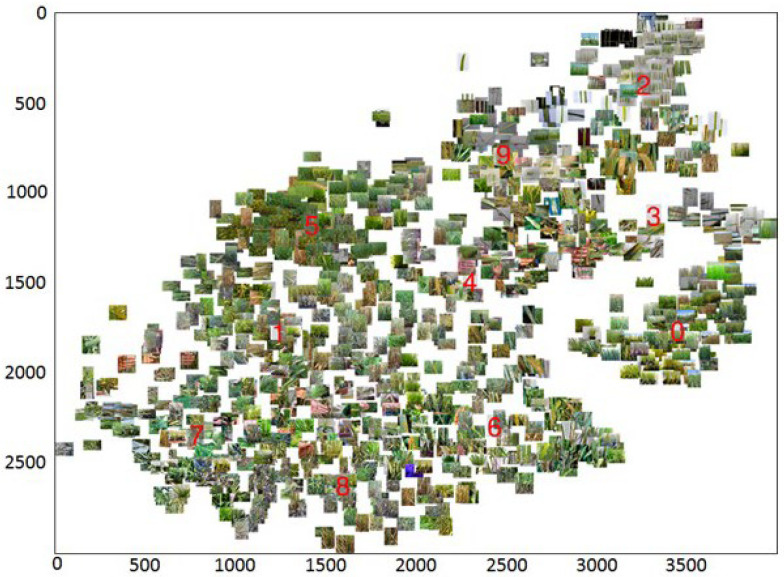
The distribution of 1000 randomly chosen images in the space of two components obtained by using the t-SNE dimensionality reduction method for low-frequency components of Fourier spectra of images. Ten clusters, from 0 to 9, shown by red numbers.

One of the strategies of the analysis, EfficientNet-B0_FDA, implied a procedure for transferring image styles in the training set during the augmentation process. In order to select the characteristic image styles in the sample based on the low-frequency part of their Fourier spectra, we classified the styles as described in the “Training Strategies” section for 1000 randomly selected training sample images. The results are presented in Figure 6. This diagram shows the distribution of images in the space of two components obtained by using the t-SNE dimensionality reduction method for low-frequency components of Fourier spectra of images. The distribution of images on the diagram is non-uniform, and a number of clearly visible clusters can be distinguished in it. Clustering images in the space of these two components by the *k*-means method allowed us to obtain 10 characteristic image styles, the centroids of which are indicated in Figure 4 by the corresponding numbers. In particular, Cluster 5 corresponds to images of wheat in plots in the field conditions. Cluster 2 includes the images obtained under laboratory conditions for leaves affected by diseases, in Petri dishes. In the center of the diagram (clusters numbered 4, 1 and 7), there are images containing hands. Thus, the sample of images was heterogeneous in their styles, since the images acquired by using different protocols were used to obtain it. The 10 characteristic types of styles identified by cluster analysis were further used in the training process according to the EfficientNet-B0_FDA strategy.

### 3.4. Assessment of Image Classification Accuracy

**Figure 7:**
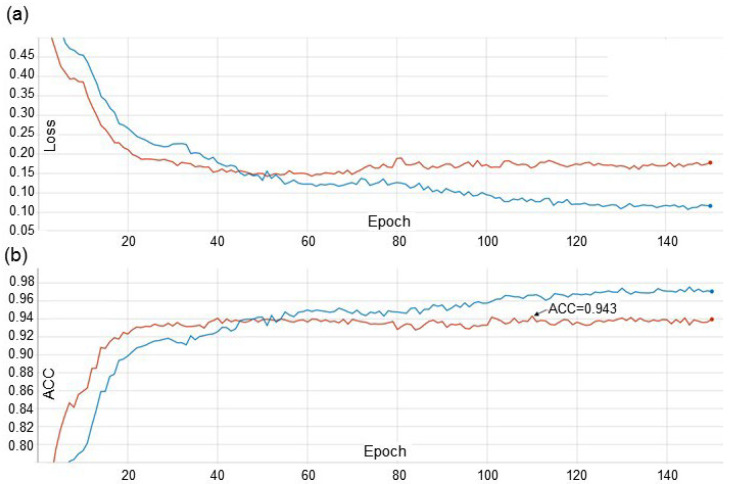
Changes in the loss function (**a**) and ACC accuracy (**b**) from the training epoch of the EfficientNet-B0_FDA model. Blue line—train dataset; red line—validation dataset.

An example of the learning curves obtained for the EfficientNet-B0_FDA strategy are shown in Figure 7. It can be seen from the figure that, for the chosen number of epochs, 150, the Loss and ACC values for the training and validation data become stationary. The ACC values after becoming stationary for the validation sample are within 0.92–0.94, reaching a maximum value of 0.943 at epoch 110. The network parameters obtained for this epoch were used for further testing on the hold-out sample; the accuracy was 0.942 (see Table 5). The difference in accuracy between validation and deferred samples was 0.1%, which indicates that there is no overfitting effect.

For the EfficientNet-B0_baseline model, the stationary values of Loss and ACC were reached already at Epoch 10, and the optimal parameters were obtained for Epoch 18 (Table 5). On the test sample, the accuracy of this network reached 0.933. For the EfficientNet-B0_augm strategy, it became stationary at Epoch 60, and the optimal parameters were obtained for Epoch 140. The accuracy of this model on the test sample was 0.939 (Table 5).

From the data presented in Table 5, it can be concluded that the use of augmentations in the training process increases the accuracy on deferred sampling by 0.6% (EfficientNet-B0_augm vs. EfficientNet-B0_baseline). The addition of the FDA style transfer to the augmentations increases the image recognition accuracy by another 0.3% (EfficientNet-B0_FDA vs. EfficientNet-B0_augm). For the EfficientNet-B0_FDA model, we additionally evaluated the disease prediction accuracy for seedlings and adult plants separately. In the first case, the ACC value was 0.963, and in the second one, 0.926.

The recognition accuracy of individual labels for images of the test sample is presented for all three strategies for training the network in Table 6. It shows that the improvement in accuracy when changing the strategy for different labels does not always change systematically depending on the strategy. The increase in accuracy with the increasing complexity of the strategy (EfficientNet-B0_baseline -> EfficientNet-B0_augm -> EfficientNet-B0_FDA) is typical for disease-related labels, but it decreases slightly when identifying healthy plants and seedlings. The highest recognition accuracy among disease labels is achieved for “septoria” (0.956), and the lowest one is for “leaf rust” (0.890).

The performance characteristics for different labels for the EfficientNet-B0_FDA neural network training strategy are shown in Table 7. In terms of the *F1*-score, the best performance is achieved for seedling recognition. The lowest value is for the septoria (0.686); other disease types have an *F1* score between 0.815 and 0.852.

**Figure 8:**
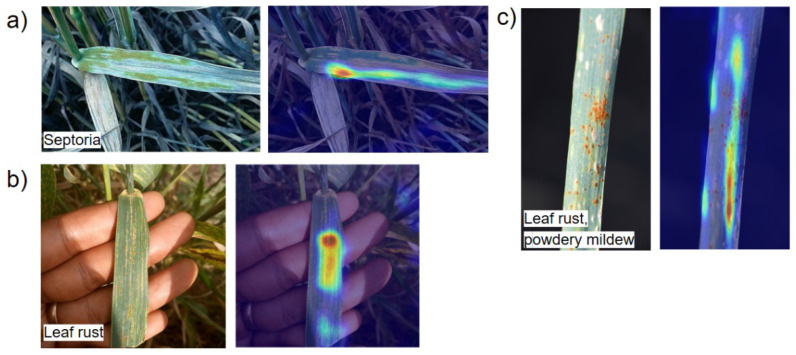
Visualization of the activation maps for three images with disease symptoms by Grad-CAM: (**a**) septoria, (**b**) leaf rust, and (**c**) leaf rust and powdery mildew. The original image is shown in the left part of the panel, and the activation map diagram is shown on the right part of the panel. The level of the activation is shown by color from low (blue) to high (red) values.

Examples of the activation map for the images of plants with disease symptoms are shown in Figure 8. The figure demonstrates that the focus in the image is at the disease lesions located in the green parts of the leaves, not at the dead leaves (Figure 8a, septoria as an example). Our network discriminates well between plant disease lesions and human hands, despite their similar color, as demonstrated in the Figure 8b (leaf rust as an example). For the complex diseases (Figure 8c, leaf rust and powdery mildew, as an example), the network is able to detect, at least partially, both the leaf rust pustules and powdery mildew grayish white colored patches on the leaf.

**Figure 9:**
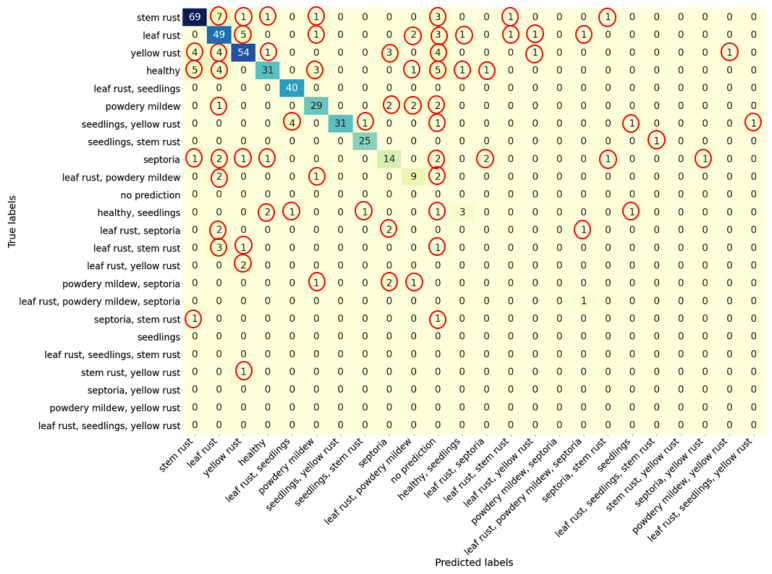
Confusion matrix for the test sample for the EfficientNet-B0_FDA model. Rows, true labels; columns, predicted labels. The off diagonal cells with non-zero values are highlighted by red circles.

A confusion matrix for identifying labels in images for the EfficientNet-B0_FDA model, built on the basis of test sample, is shown in Figure 9. The diagonal elements of the matrix are significantly larger than the off-diagonal ones, which characterizes the high accuracy of image classification by our method. An analysis of the matrix shows that the largest fraction of misclassifications among rust diseases falls on other rust diseases. Thus, for the label “stem rust”, 69 images (82%) are classified correctly; four images are classified as “yellow rust”, and five images as “healthy”. For the label “leaf rust”, 49 images (77%) are classified correctly, seven images are classified as “stem rust”, four images as “yellow rust”, and four images as “healthy”. For the “yellow rust” class, 54 images (75%) are classified correctly, five images are incorrectly classified as “leaf rust”, and one image as “stem rust”. A slightly smaller, but noticeable part of the images from these classes appeared in the “no prediction” class. Another type of error for these labels was placing them into the categories with multiple labels of rust diseases.

**Figure 10:**
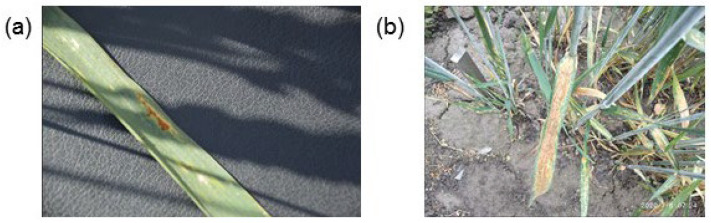
Examples of images marked with several disease labels at the same time, for which one of the diseases is not recognized. (**a**) Image 00c0f87c20f0fcff.jpg: labels “leaf rust” (identified) and “powdery mildew” (not identified). (**b**) Image 0dedf8d8db703948.jpg: labels “leaf rust” (not identified) and “septoria” (identified).

In the case of images of plants affected by several pathogens, we observed that sometimes one of the pathogens is predicted correctly, but the symptoms of the second pathogen are recognized with less accuracy due to the low value of the corresponding output neuron. For example, for the class “leaf rust, powdery mildew”, nine images are classified correctly, two images are classified as “leaf rust”, two images as “powdery mildew”, and one image as “powdery mildew, septoria”. An example of such an image classified by the expert as “leaf rust, powdery mildew” is shown in Figure 10a. In the center of the leaf, an area affected by leaf rust is clearly visible, and two spots affected by powdery mildew are visible on the left above and on the right below. However, the area on the right below is blurred due to the lack of focus of the camera. This is likely the reason why the label “powdery mildew” was not identified.

For the multiple label “leaf rust, septoria”, no images were predicted correctly; however, out of five such images, four were assigned to individual labels: two to “leaf rust” and two to “septoria”. An example of one such image is shown in Figure 10b. It shows the shoots strongly affected by septoria, especially the leaves in the foreground of the image. Against such a background, symptoms of leaf rust damage are barely noticeable (the plant is in the upper left corner of the frame near the peg).

Another example is the definition of joint labels “leaf rust, powdery mildew, septoria”; one image is classified correctly, one image as “leaf rust”, and one image as “leaf rust, septoria” (Figure 9).

It is interesting to consider the case of classifying such a label as “seedling”. It is noted that such a label was not separately presented in the labeling of any image. This label has always been found either together with the label of some disease or with the label of a healthy plant (see Table 4). The analysis of the confusion matrix shows that, in most of all test images in which this label was present, our algorithm predicted it. This gives an accuracy of 0.959 for the “seedling” plant type. In several cases, the model assigned the label “seedlings” to images that were not tagged with such a label. In two cases, the “seedlings” label was predicted to be the only one (for the “healthy seedlings” and “seedlings, yellow rust” labels), but it should be noted that in these two cases the plants in the images were seedlings.

It should be noted that, if no weight is determined for any of the labels greater than the specified threshold of 0.5, the model returns the label “no prediction” (absence of classification). This label was assigned to 25 images (5%). The largest share of such classifications was obtained for the label “healthy” (16%).

### 3.5. Bot in the Telegram Messenger

**Figure 11:**
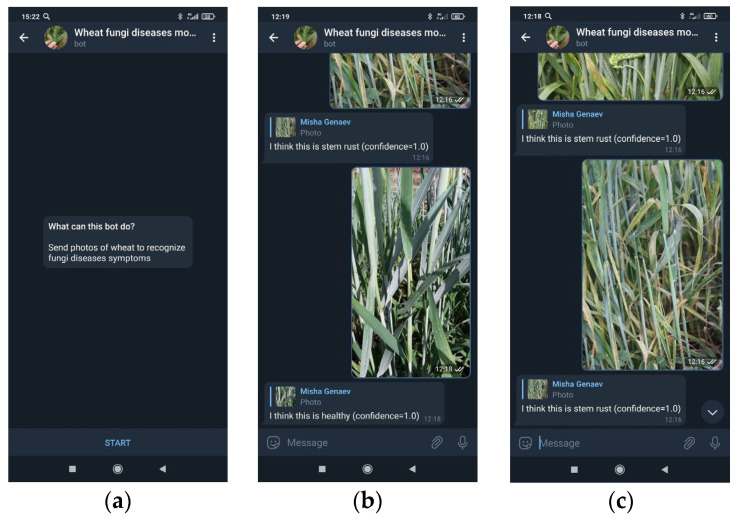
User interface for interacting with the bot @wheat_healthy_bot in the Telegram messenger: (**a**) begin dialogue; (**b**) example of image classification, healthy plant; and (**c**) example of image classification, stem rust.

The proposed recognition model was implemented for use as the bot @wheat_healthy_bot in the Telegram messenger https://t.me/wheat_healthy_bot (accessed on 16 July 2021). The Telegram application is available for all popular platforms, which allows us to use this service both in the field conditions via mobile devices and in the laboratory, using a stationary computer. The user can send the bot an image of a plant and receive a prediction of its state (damage by a disease and/or developmental stage). This service allows users to identify plant diseases by using mobile devices in field conditions. The user interface for interacting with the bot @wheat_healthy_bot is shown in Figure 11.

## 4. Discussion

In this paper, a method was proposed for identifying wheat diseases based on digital images obtained in the field conditions and the algorithm based on the EfficientNet-B0 architecture network.

It should be noted that a sample of well-annotated images for training and testing is one of the important conditions for creating a successful method of disease recognition [50]. Unfortunately, the existing open databases of images of plant diseases contain few images of wheat. For example, there are nine of them in the PDDB database [34], but they are absent in the well-known PlantVillage dataset [53]. For the analysis of wheat diseases, in the work by Picon et al. [48], the sample included 8178 images (according to the imaging protocol, these data contained images of individual leaves, but not the whole plant). In the work by Lu et al. [49], a sample of Wheat Disease Database 2017 (WDD2017) was collected from 9230 images of not only leaves but also plants in field conditions. Arsenovich et al. [54] used a dataset that included information on 5596 images of wheat (the authors do not provide details). These three abovementioned sets, however, are not freely available. Therefore, in our work, based on several open sources and our own data, a set of wheat images was formed both in the field and in the laboratory conditions. It is important to note that, in our sample, images from external sources were relabeled by phytopathologists for six types of diseases, both separately and jointly. The WFD2020 data are freely available for downloading.

One of the features of the sample is a special class of images for seedlings. Special attention was paid to this, since the timely diagnosis of the harmful complex in the early periods of the vegetational season is of particular importance. Under favorable weather conditions, diseases can develop rapidly and lead to significant losses of yield. Therefore, to obtain large yields, it is important to monitor the lesion of flag and pre-flag leaves and diagnose the type of pathogen of the disease, which makes it possible to select effective means of protection [83]. As a result of the research, it appeared that the accuracy of disease prediction is higher for seedlings. However, this may also be related to the dataset peculiarities: the variety of shooting conditions for seedlings is less than when shooting adult plants.

The accuracy of determining diseases by using the developed method for the most optimal learning strategy (EfficientNet-B0_FDA) was 0.942 which is comparable to the results of other authors. For example, Picon et al. [48] used a network architecture based on ResNet50 to identify lesions of septoria, tan spot, or rust (either leaf or yellow). The accuracy of disease identification was 97%. Lu et al. [49] tried several options of neural networks for deep learning and multiple instance learning to identify wheat lesions by the six most common diseases. The performance of our approach is comparable to the accuracy of methods for assessing plant diseases not only for wheat but also for other crops. The recognition accuracy ranged from 95 to 97% for plant-disease symptoms in Reference [54]; on the basis of the PlantVillage dataset and the authors’ data, a two-stage network architecture was developed, which showed an average accuracy of disease recognition of 0.936. In Reference [60], the problem of simultaneous identification of 14 crop species and 26 diseases was also solved on the basis of the PlantVillage dataset. The network of the ResNet50 architecture was used, which achieved an accuracy of 99.24%.

It is interesting to compare our results with the accuracy obtained for plant-disease recognition from using similar architecture, EfficientNet. Atila et al. [42] evaluated the performance of all eight topologies of this family on the original PlantVillage dataset and demonstrated that the highest average accuracy in disease recognition was achieved for the B5 architecture (99.91%). However, all models of the family obtained average accuracy very close to each other.

Zhang et al.’s work [41] is very similar to ours. They predicted the disease symptoms for a single crop, cucumber, on the images taken in a naturally complex greenhouse background. They detected three types of labels (powdery mildew, downy mildew, and healthy leaves) and combination of powdery mildew and downy mildew. Their dataset included 2816 images, which is close to our dataset size. They used different optimization methods and network architectures and obtained the best accuracy for the EfficientNet-B4 network and Ranger optimizer, 96.39% on the test sample. Interestingly, the EfficientNet-B0 architecture demonstrated accuracy of 94.39% on this dataset [41], which is very close to our results (94.2%).

It should be noted that the difference in the accuracy of our method between the validation and test datasets is small (0.1%), which indicates the overfitting absence for our model. This is not surprising, because we used the EfficientNet architecture with the smallest number of parameters (B0) and the strategy of the dataset stratification.

Our analysis of the matrix of errors showed that their main sources were the incorrect classifications of diseases, for example, rust ones, among themselves. Interestingly, the cross-misclassification between rust diseases and the other two (septoria and powdery mildew) was found to be higher for septoria. This is explainable since the visual symptoms of these diseases can be very similar. In addition, for some images, the prediction of the pathogen type was not reliable enough to perform classification (“no prediction” result).

The possibility of using the technology of identification of crops by pathogens in the field is one of the necessary properties of the system for operational monitoring of diseases. In this direction, methods are being actively developed that integrate the results of prediction by the method of deep learning and the implementation of access to them via smartphones and tablets [48,49,61,62]. This, however, requires additional effort to build and maintain mobile applications. Here, we took advantage of the possibility of a simple interface through the use of a Telegram messenger bot. Recently, this type of application has become popular and is used for crowdsourcing problems in social research [84], for predicting real-estate prices [85], for determining the type of trees [86], etc. Its advantages are that there is no need to develop and maintain a smartphone graphical interface, and, at the same time, that it allows us to send an image as a request to a server for further processing and displaying the results. At the same time, access to the service is possible wherever there is access to the Internet both from a mobile device and from a desktop PC.

## 5. Conclusions

The paper proposed a method for recognizing plant diseases on images obtained in field conditions based on the deep machine learning. Fungal diseases of wheat as leaf rust, stem rust, yellow rust, powdery mildew, septoria, and their combinations were recognized. Additionally, the network determines whether the plant is a seedling. The image dataset represents a specially formed and labeled sample of 2414 images, which is freely available.

The algorithm is based on the EfficientNet-B0 neural network architecture. We implemented several techniques to achieve better performance of the network: transfer learning; dataset stratification into training, validation and testing samples; and augmentation, including image style transfer.

Of the three examined learning strategies, the best accuracy was provided by the method using augmentation and transfer of image styles (the accuracy was 0.942). These results are comparable with the performance obtained by other deep learning methods for plant-disease recognition. Our network performance is in good agreement with the results obtained by implementation of the EfficientNet architectures for plant-disease recognition.

The interface to the recognition method was implemented on the basis of the Telegram chatbot, which provides users with convenient access to the program via the Internet, both from a mobile device and using a desktop PC. This allows users to utilize our recognition method for wheat plants in the field conditions by using the images obtained via smartphone camera under real-world circumstances.

## Figures and Tables

**Table 1:** Sources of images in the dataset Wheat Fungi Diseases.

Source	Number of Images	Reference
Zindi.africa	874	[63]
Google Images	259	-
PDDP	121	[61,64]
Saint Petersburg	367	This work
Novosibirsk	793	This work

**Table 2:** Parameters of the EfficientNet-B0 network used in our work.

Stage	Operator	Resolution	Number of Channels	Number of Layers
1	Conv3 × 3	512 × 512	32	1
2	MBConv1, k3 × 3	256 × 256	16	1
3	MBConv6, k3 × 3	256 × 256	24	2
4	MBConv6, k5 × 5	128 × 128	40	2
5	MBConv6, k3 × 3	64 × 64	80	3
6	MBConv6, k5 × 5	32 × 32	112	3
7	MBConv6, k5 × 5	32 × 32	192	4
8	MBConv6, k3 × 3	16 × 16	320	1
9	Conv1 × 1 and Pooling and FC	16 × 16	1280	1

**Table 3:** The number of labels of each class in the WFD2020 dataset.

Class	Number of Labels (%)
leaf rust	655 (20.8%)
stem rust	591 (18.8%)
yellow rust	571 (18.1%)
powder mildew	277 (8.8%)
septoria	185 (5.9%)
seedlings	569 (18.1%)
healthy	299 (9.5%)
Total	3147 (100%)

**Table 4:** The number of images with different combinations of labels in the WFD2020 dataset.

Class	Number of Labels
stem rust	426 (17.6%)
yellow rust	362 (15.0%)
leaf rust	334 (13.8%)
healthy	253 (10.5%)
leaf rust, seedlings	198 (8.2%)
seedlings, yellow rust	197 (8.2%)
powdery mildew	187 (7.7%)
septoria	133 (5.5%)
seedlings, stem rust	127 (5.3%)
leaf rust, powdery mildew	65 (2.7%)
healthy, seedlings	46 (1.9%)
leaf rust, stem rust	20 (0.8%)
leaf rust, septoria	17 (0.7%)
powdery mildew, septoria	12 (0.5%)
leaf rust, powdery mildew, septoria	11 (0.5%)
septoria, stem rust	11 (0.5%)
leaf rust, yellow rust	7 (0.3%)
stem rust, yellow rust	2 (0.1%)
powdery mildew, stem rust	2 (0.1%)
leaf rust, stem rust, yellow rust	2 (0.1%)
septoria, yellow rust	1 (0.0%)
leaf rust, seedlings, stem rust	1 (0.0%)
Total	2414

**Table 5:** The value of the target metric of the model accuracy on the validation and test sample for different model training strategies.

Model Name	Number of Epochs	ACC Valid	ACC Test
EfficientNet-B0_baseline	18	0.938	0.933
EfficientNet-B0_augm	140	0.941	0.939
EfficientNet-B0_FDA	110	0.943	0.942

**Table 6:** Accuracy of determining various types of labels on the test sample for the three used neural strategies of neural network training.

Label	EfficientNet-B0_Baseline	EfficientNet-B0_Augm	EfficientNet-B0_FDA
leaf rust	0.881	0.881	0.890
stem rust	0.910	0.925	0.929
yellow rust	0.927	0.915	0.931
powdery mildew	0.940	0.952	0.954
septoria	0.927	0.952	0.956
seedlings	0.990	0.990	0.988
healthy	0.933	0.958	0.938

**Table 7:** Performance characteristics for the EfficientNet-B0_FDA neural network training strategy.

Label	Precision	Recall	*F1*-Score
leaf rust	0.803	0.852	0.827
stem rust	0.892	0.816	0.852
yellow rust	0.920	0.748	0.825
powdery mildew	0.830	0.800	0.815
septoria	0.770	0.619	0.686
seedlings	0.982	0.960	0.971
healthy	0.824	0.615	0.704

## Data Availability

The WFD2020 dataset is available at http://wfd.sysbio.ru/ (accessed on 16 July 2021). Telegram bot for disease recognition is accessible at https://t.me/wheat_healthy_bot (accessed on 16 July 2021). The network model with example script is accessible at http://wfd.sysbio.ru/data/model_and_example.zip (accessed on 16 July 2021).
